# The future of births via medically assisted reproduction in Italy:Scenarios to 2050

**DOI:** 10.1371/journal.pone.0354135

**Published:** 2026-07-21

**Authors:** Alessandra Burgio, Cinzia Castagnaro, Gustavo De Santis, Daniele Vignoli, Agnese Vitali

**Affiliations:** 1 Italian National Institute of Statistics, Rome, Italy; 2 Department of Statistics, Computer Science, Applications, University of Florence, Florence, Italy; 3 Department of Sociology and Social Research, University of Trento, Trento, Italy; IULM: Libera Universita di Lingue e Comunicazione, ITALY

## Abstract

Delayed childbearing is increasingly common in high-income societies, contributing to rising demand for medically assisted reproduction (MAR). This paper presents nine possible scenarios of the future share of MAR births on the total number of births in Italy, a country internationally known for its latest-late mean age at childbearing. We combined the latest (2023) official data and three hypotheses on the evolution of maternal mean age at childbirth (no change, slow ageing, rapid ageing) with three hypotheses on the age-specific MAR-related fertility rates (no change, moderate increase, rapid increase). Our projections indicate that changes in the age structure of women of reproductive age will likely have a negligible effect on future MAR prevalence. In contrast, delayed childbearing and continued expansion of MAR use could raise the share of MAR births from 4.3% in 2023 to 11–12% by mid-century, with a plausible upper bound exceeding 15% under sustained trends. These results underscore the growing demographic, social and economic significance of MAR in Italy and highlight important implications for public health planning, resource allocation, and policies aimed at supporting earlier childbearing.

## Introduction and purpose

Delayed reproduction is increasingly common worldwide, particularly in Europe [1], and especially in Italy [2]. The causes are multifaceted [3]. Extended periods of education [4], high youth unemployment [5], and various forms of uncertainty—related to income, women’s societal roles, couple stability, and employment-linked residential choices [6,7]—all contribute to postponement, alongside the perception that “there will be time to catch up later” [8,9]. Nonetheless, this catch-up mechanism does not always operate: completed fertility often falls short of intentions, sometimes resulting in involuntary childlessness [4,8,10]. Biological constraints, including declining fecundity with age [11,12], infertility [2] and increased pregnancy-related risks—such as miscarriages and stillbirths [13,14]—further limit the capacity to recover fertility later in life.

In this context, medically assisted reproduction (MAR) techniques are playing an increasingly important role. MAR is used here in its broad sense, encompassing not only assisted reproduction treatments (ART) such as in vitro fertilization (IVF), intracytoplasmic sperm injection (ICSI), preimplantation genetic testing, and cryopreservation of embryos and gametes, but also assisted insemination and hormonal treatments such as ovulation induction or stimulation [15]. MAR techniques serve both as a remedy, helping couples overcome obstacles and achieve their desired family size, and as a potential driver of delayed fertility, by making late childbearing appear more feasible—an ambivalent effect noted in several studies [16]. At the same time, infertility remains a major reproductive health issue affecting both women and men, although male infertility continues to be underrecognized in both research and policy discussions [17,18].

Most research on the expanding medicalization of reproduction focuses on ART, largely due to data availability [16,19,20], with a few exceptions such as Burgio et al. [2]. These studies generally examine past trends, current levels [21], and group differences—e.g., by educational attainment or socioeconomic status [5,22]—while only a few attempt to project trends in MAR use into the future, e.g., Sobotka et al. [23], Tierney [19], and Lazzari et al. [24].

This paper aims to extend these analyses by providing plausible projections of MAR-related births (not limited to ART) in Italy up to 2050, based on the most recent official data and scenarios consistent with the latest population and fertility projections from the Italian National Institute of Statistics (Istat). This is not merely a statistical matter, for two reasons. First, the role of MAR is growing worldwide, particularly in affluent countries with low and delayed fertility, among which Italy is a leading example. Second, there are interesting context-specific developments: infertility was officially recognized as a medical condition in Italy in 2024, and since January 2025, MAR treatments have been included in the national “essential levels of care” (LEA), greatly improving accessibility and affordability. For a comprehensive overview of the legislation governing MAR in Italy, see Burgio et al. [2].

These reforms have the potential to boost MAR uptake in Italy over the coming decades, but the magnitude of this effect is unclear, in part because several constraints remain, both practical and legal. Specialized centers are still relatively few, especially in the south of the country. Besides, services provided under LEA are limited to heterosexual co-residing couples, with the woman younger than 47 years, and restricted to homologous fertilization (using the couple’s own gametes) at a modest fee (~€300). Heterologous fertilization (using donor gametes) is also available under LEA at a participation fee of ~€1,500, which is about one-third of the cost charged by private clinics.

## Materials and methods

### Data

This study relies on a combination of administrative data covering the entire population of live births in Italy in 2023, the latest date available at the time of writing. We use the Birth Delivery Certificate (CeDAP) administered by the Ministry of Health and two Istat sources: the Register of live births referred to residents and the Register of resident population by age and sex, to identify women at risk of having a (further) child. The shares of medically assisted reproduction on live births derive from CeDAP data on MAR live births by mother’s age: a reweighting procedure, based on the Register of live births data, ensures consistency with official demographic statistics.

Age-specific fertility rates were calculated separately for births conceived via medically assisted reproduction (MAR) and those conceived naturally. For further details on the data sources and the calculation of MAR and non-MAR fertility measures, we refer readers to Burgio et al. [2].

**Fig 1:**
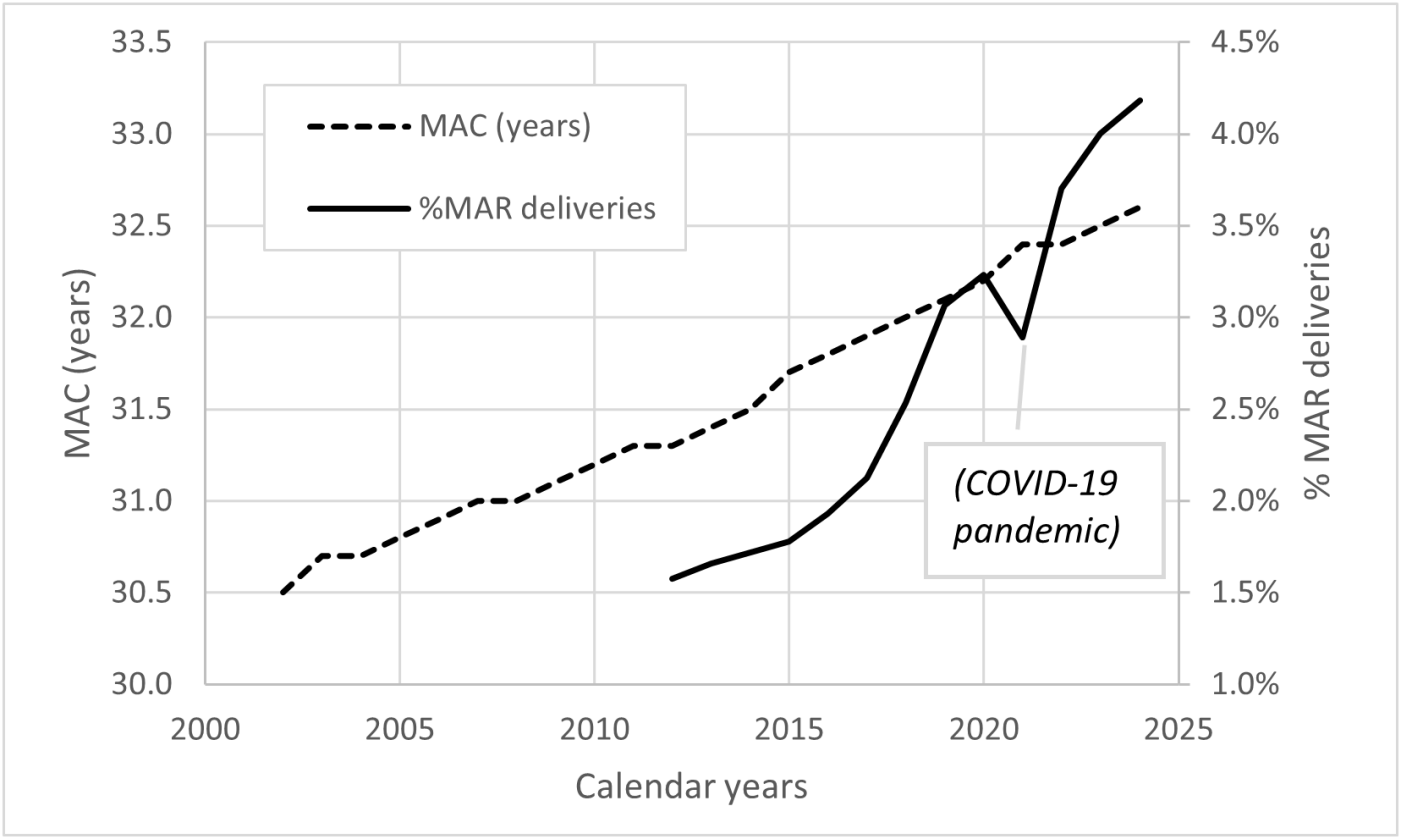
Mothers’ mean age at childbirth (MAC), 2002–2024 and share of medically assisted deliveries, 2012–2024, Italy. Source: Istat and Ministry of Health.

At a glance, MAR contributed 3.9% to Italy’s average number of children per woman—or total fertility rate (TFR)—in 2023, up from 2.1% in 2013. MAR births were substantially more frequent among older mothers: 17.2% for women aged 40 years and over in 2023 (8.6% in 2013). The mean age at first birth among MAR births increased from 36.0 years to 37.7 years between 2013 and 2023, compared to 30.4 years and 31.4 years for naturally conceived births over the same period, highlighting MAR’s growing association with delayed childbearing (Fig 1).

### Projection method

We projected population and births to 2050, taking 2023 as the base year. We designed nine future scenarios, stemming from a combination of projections based on the cohort-component method [25], a standard demographic technique that uses age- and sex-specific input data to project a population into the future, breaking it down by age and sex. Projections are carried out incrementally, typically over five-year intervals. At each step, the method first projects each age cohort according to survival rates, adjusts for net migration during the interval, and then calculates the number of children born, whose expected mortality and migration are later also taken into account. This iterative process allows for a detailed and dynamic representation of population change over time.

In our case, we decided to adhere as closely as possible to the existing official (Istat) population and birth projections, which were carried out using the same cohort-component method. We complemented our results with a set of derived projections, to estimate outcomes for additional variables of interest. Derived projections apply independently estimated rates or probabilities to the age- and sex-specific population obtained from the cohort-component method. In our study, we imposed scenarios combining different levels of change in (a) the timing of childbearing and (b) the uptake of MAR procedures. The resulting estimates provide the expected proportion of MAR births under each scenario, conditional on the population dynamics already captured by the cohort-component framework.

### Starting points

Our initial year is 2013 because it marks the first year in which reasonably reliable—though not perfect—information on MAR is available with satisfactory coverage. The most recent year considered in our study is 2023, the latest available at the time of writing. For both 2013 and 2023, we have data on general fertility and MAR-specific fertility rates by maternal age. Although information on birth order is also available, we did not use it here, as our study applies observed and projected proportions of MAR fertility to official (Istat) future fertility rates, which are not disaggregated by birth order.

As mentioned, we endeavored to reproduce as closely as possible Istat population projections, published in July 2025 [26]. These projections start in 2024 and extend to 2080, although, for the purposes of this paper, we stopped earlier: 1st January 2050 for population stocks and 2045–2049 for flows, including fertility.

As explained below, to project MAR’s future contribution to fertility, age-specific fertility rates (fₓ,ₜ) for each age group x and year t are needed. These rates, however, are not provided by Istat and therefore must be reconstructed. Similarly, we used official life expectancy and migration projections (net flows), which also lack age-specific detail, necessitating reconstruction.

In other words, we computed our own population projection of the Italian population and births up to 2050: these align closely with, but are not identical to, the official projections. All divergences, however, are of little practical consequence for our primary interest: the evolution of the share of births attributable to MAR.

### Mortality and migration hypotheses

Istat provides information on life expectancy (for survival) and total international migration rates without any age-specific breakdown. To address this, we applied standard age schedules to allocate rates and net migrants across age groups. For mortality, we used Brass’ method [27] to reconstruct a survival curve consistent with the Istat’s projected life expectancy at birth, which increases from 85.4 years in 2024 to 87.5 years in 2045–2049 for females.

For migration, we employed Rogers and Castro’s [28] age schedules to redistribute the expected number of net immigrants (approximately 3 per 1,000 population per year, as hypothesized by Istat) across age groups.

In both cases, we do not delve into the technicalities of the procedures for two reasons. First, because both are consolidated demographic techniques. Second, and most importantly, because both merely redistribute events, or rates, in such a way to smooth curves, so that their age-related evolution makes sense, and is consistent with observed data, or with the constraints of the problem at hand (e.g., a given improvement in survival).

### Hypotheses about fertility and share of MAR births by mother’s age: generalities

**Fig 2:**
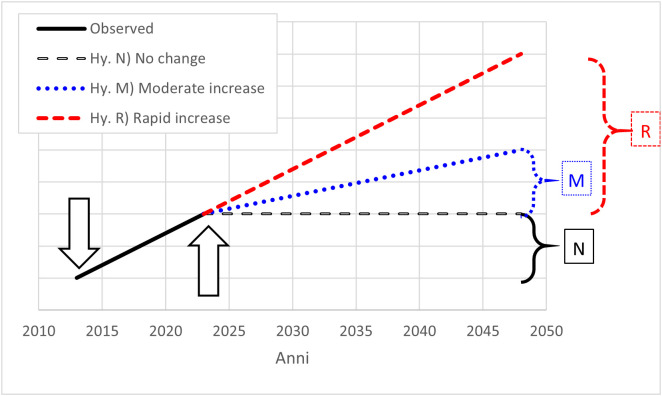
Schematic representation of the evolution observed between 2013 and 2023 and foreseen in the future, up to 2045–2049, according to three hypotheses on two variables, MAC and %MAR. Note: Arrows represent observed years, 2013 (downwards) and 2023 (upwards). Hypotheses are: N) No change; M) Moderate increase; R) Rapid increase. In all three cases, and for both variables, the change is hypothesized to be (practically) linear over the next 25 years. MAC: mothers’ mean age at childbirth; %MAR: share of MAR births on total births by mother’s age.

We constructed nine scenarios, combining three hypotheses on the future evolution of two different variables: the mean age at childbirth (MAC) and the age-specific MAR-related fertility rates (%MAR). While all scenarios are presented in detail in the following sections, here we briefly illustrate the general logic underlying our approach, which applies in both cases. Both variables increased almost linearly in the latest observable years (Fig 1), and we expect this tendency to continue in the near future. We expect age at delivery to continue increasing because its underlying socio-economic processes—e.g., gender equality, educational expansion, postponed transition to adulthood, employment- and housing-related uncertainties—are still at work. With regard to the share of MAR births, future increases will probably be driven by gradual improvements in access, treatment effectiveness, and social acceptance. We hence assumed that possible evolutions for our two key variables, MAC and %MAR, will continue to follow a linear trend also in the future. We developed three hypotheses for each of our key variables over the next 25 years. These three hypotheses are reported in Fig 2 and are indicated with the symbols N, M, and R:

N) No change: no yearly increase of MAC (%MAR) in the next 25 years compared to the base year (2023). This is the status-quo scenario, useful as a standard of reference.
M) Moderate increase: the absolute increase of MAC (%MAR) in the next 25 years is assumed to be the same as the one observed in the decade 2013–2023. Given the different length of the time interval, under the M hypothesis, the yearly increase in the next 25 years is projected to be smaller than the one observed during the decade 2013–2023.
R) Rapid increase: the same yearly absolute increase of MAC (%MAR) in the next 25 years as observed in the decade 2013–2023.

We do not consider hypotheses involving future decreases in MAC or %MAR as we deem such scenarios highly unlikely in the medium-long term. We acknowledge, however, that the %MAR may be susceptible to short-term declines, as it happened, for example, during the COVID-19 pandemic (Fig 1). Similarly, a decline in %MAR is possible during 2025, due to the difficulties faced by (some) Italian regions in absorbing the increased demand for treatment, following the introduction of the new, more permissive law including MAR among LEA treatments. Such variations, however, are expected to be limited to a few specific years, leaving long-term trends unaffected.

### Hypotheses on the fertility schedule

Our projected TFRs for the period 2025–2049 are the same as Istat’s, rising slowly from 1.21 at the beginning of the period to 1.37 at the end. However, Istat did not disclose information on the age-specific fertility rates it used, and this is essential for our analysis, as the use of MAR tends to increase with maternal age. We know, nonetheless, that fertility in Italy has been progressively delayed over the past five decades, with the mean age at childbirth (MAC) increasing almost linearly from 30.5 years in 2002 to 32.6 years in 2024 (Fig 1).

**Fig 3:**
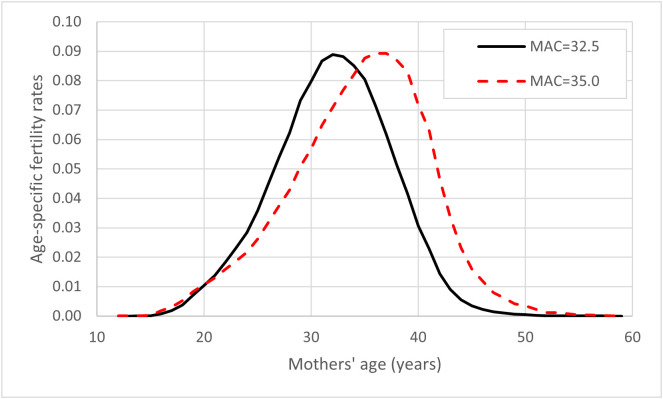
Age-specific fertility rates fₓ in Italy, 2023 (observed, with MAC = 32.5 years) and projected to 2045–2049 under the rapid fertility ageing scenario (with MAC = 35.0 years). Notes: MAC: Mothers’ mean age at childbirth. An age-specific fertility rate fₓ is calculated as Bₓ/Wₓ, where x = age, Bₓ = births to mothers aged x, and Wₓ = women aged x. Projections done with Brass’ method [27]. The case where MAC = 32.5 years was observed in 2023 and corresponds to our N (no change) hypothesis. The case where MAC = 35.0 years corresponds to our R (rapid increase) hypothesis. Our M (moderate increase) scenario, where MAC = 33.5 years, not drawn in the figure, is intermediate between the two curves. Source: Istat and own calculations.

Based on these trends (and the same TFRs hypothesized by Istat) and given the constraints (no information on age-specific fertility rates from official statistics), we applied Brass’ method [27] to reconstruct plausible age-specific fertility distributions. We developed three scenarios for the shape of age-specific fertility rates, as follows:

N) No change: age distribution of fertility rates remains as observed in 2023 (MAC = 32.5 years; black continuous curve in Fig 3).
M) Moderate increase: slow ageing of women’s fertility schedule. MAC reaches 33.5 years by 2045–2049 (not directly shown: the resulting curve is intermediate between the two displayed in Fig 3).
R) Rapid increase: MAC increases at the same rate as in the preceding decade, reaching 35.0 years in 2045–2049 (red dashed line in Fig 3).

### Hypotheses on the future share of MAR births by mother’s age

**Fig 4:**
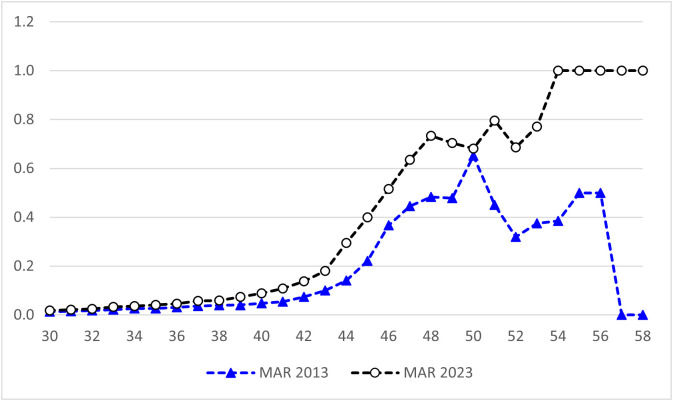
Age-specific shares of MAR births out of total births observed in Italy in 2013 and 2023. Note: Unadjusted shares. Source: Istat and Ministry of Health.

The observed shares of MAR births out of the total number of births by maternal age in 2013 and 2023 are presented in Fig 4.

Because the chances of conceiving naturally decrease with age, especially past 40 years, the proportion of MAR births out of total births should increase monotonically with maternal age, approaching 100% at very high reproductive ages, e.g., 55 years. This increase, however, is not consistently observed in the official data, likely due to underreporting of MAR conceptions by respondents (i.e., puerperal women), especially in 2013, when the practice was still rare and surrounded by limited knowledge and stigma.

As a first step, we adjusted both curves to obtain a smoother and presumably more accurate estimate of the share of MAR births by mothers’ age for 2013 and 2023. This allowed a more precise assessment of the presumed increase of the phenomenon over the decade.

**Fig 5:**
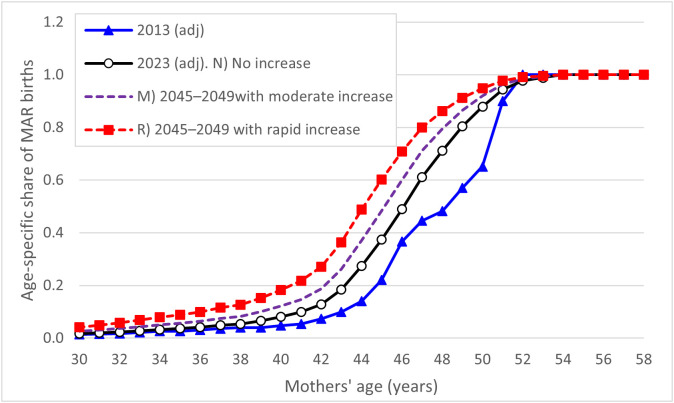
Age-specific shares of MAR births out of total births estimated in Italy in 2013 and 2023 (both adjusted) and projected to 2045–2049 in three scenarios: no increase (N), moderate increase (M), and rapid increase (R). Source: Istat and Ministry of Health.

Next, we used the adjusted 2023 curve as a reference standard, modified it using the Brass technique [27], and projected three potential evolutions in age-specific MAR shares, mirroring the approach used for MAC. Our three hypotheses on the possible future evolutions of the age-specific shares of MAR births (%MAR), shown in Fig 5, are as follows:

N) No increase: same %MAR as in 2023 (adjusted curve).
M) Moderate increase: Over the next 25 years, the age-specific share of MAR births out of total births rises by the same amount observed in 2013–2023. Keeping the 2023 fₓ distribution constant (see Fig 3), this corresponds to a 1.8-percentage-point increase, from 2.1% (2013) to 3.9% (2023) and finally to 5.7% (2045–2049).
R) Rapid increase: This scenario assumes a continuation of the 1.8-percentage-point rise per decade, yielding a projected share of 8.4% MAR births in 2045–2049.

Three observations are in order. First, the share of MAR births calculated on the TFR does not match that observed in actual births. This discrepancy arises from differences in the age distribution of women. TFR-based calculations assume a rectangular age structure, whereas actual births reflect the observed age distribution, which may be older or younger and typically varies over time. Since Italy’s population of mothers is older than in the theoretical, rectangular case, the observed share of MAR births is higher than the TFR-based expectation.

Second, when projecting increases in MAR-attributable fertility, we hold the age schedule of fertility constant at its 2023 shape. Consequently, shifts in the fertility age profile will affect the share of MAR births. In particular, delayed fertility (leading to a higher MAC) inflates this share.

Finally, MAR births generally occur at maternal ages substantially older than those observed for naturally conceived births—approximately six years older in Italy [2]. As the proportion of MAR births grows, the effect on the average age of MAR mothers is relatively modest (Fig 5). Very high maternal ages (e.g., 50+) are minimally affected, because births occurring at these advanced ages were already predominantly MAR. As the proportion of MAR mothers increases primarily between 38 and 50 years [2], the practice remains concentrated among older women, and the average age at MAR births barely changes.

## Results

We remind readers that our projections closely mirror the latest (2025) available Istat projections [26], to which we added three hypotheses about the distribution of age-specific fertility rates: no, moderate, or rapid ageing. Within this framework, we constructed nine scenarios by combining three variants for future changes in maternal mean age at childbirth (MAC) with three variants for the age-specific proportions of births resulting from medically assisted reproduction (%MAR). In both cases, the scenarios are: N) No change (same as in 2023); M) Moderate change (same trend, but slower increase); and R) Rapid change (same trend, same pace of increase).

Our primary variable of interest is the overall share of MAR-born children among total live births. This share may, in principle, evolve over time under the influence of three separate forces:

1. Constant vs. delayed childbearing (i.e., MAC changes according to N, M, and R hypotheses).
2. Constant vs. increasing use of medically assisted reproduction (i.e., %MAR changes according to N, M, and R hypotheses).
3. Changing population age structure over the next decades, consistent with Istat’s (and our own) projections, with specific reference to potential mothers. In practice, this means that under the N/N scenario (neither MAC nor %MAR changes), the share of MAR births could still vary due to a compositional effect: if the population of women of reproductive age becomes younger (e.g., due to large migration inflows of younger foreign women or sudden increase in fertility rates in the immediate future), the share of MAR births will decline; conversely, if women of reproductive age tend to become older, the share will increase.

Our aim is to evaluate the relative contribution of each of these forces, both separately and in combination, to the overall share of MAR births among total live births in Italy over the next 25 years. The reference point is the overall proportion of MAR births (from mothers of all ages) among total live births observed in 2023, equal to 4.3%. Our synthetic results for the final projection period (2045–2049) are summarized in Table 1. Intermediate periods are not presented, as the evolution of these proportions is almost perfectly linear across all scenarios.

**Table 1:** Share of MAR births among total births projected in 2045–2049 in nine scenarios (Italy).

		%MAR (Hypotheses)		
		No increase	Moderate increase	Rapid increase
**(Hypotheses)**	**MAC**	**3.9%**	**5.7%**	**8.4%**
**No increase**	**32.5**	4.3%	6.2%	9.1%
**Moderate increase**	**33.5**	5.7%	8.0%	11.3%
**Rapid increase**	**35.0**	8.2%	11.1%	15.2%

Note: MAC = mean age at childbirth, in years. %MAR = proportion of fertility (TFR) attributable to medically assisted reproduction. No increase = same as in 2023; Moderate = moderate increase (same increase in the next 25 years as in the past 10); Rapid = rapid increase (same increase per year as observed in the past decade).

Source: Own calculations based on Istat and Ministry of Health data.

The first point to note is that population ageing alone has a negligible effect. Assuming invariance in both the age distribution of childbearing and the share of MAR (3.9% of births, calculated on the 2023 TFR), the projected share of MAR births remains at 4.3%, as in 2023. This value is slightly higher than the TFR-based 3.9%, because demographic projections suggest that the female population of reproductive age in 2045–2049 will be older than the standard used to calculate the TFR (which is “rectangular,” with the same number of women at all reproductive ages). This age-structural difference already existed in 2023, and according to Istat and our projections, it will change very little in the coming decades.

Turning now to the other two forces—delayed childbearing and increased MAR use—their effects are evident in Table 1. The impact of delayed childbearing emerges when moving down each column, i.e., holding %MAR constant. Conversely, the effect of increasing MAR prevalence by mothers’ age becomes apparent when moving horizontally in the table, i.e., keeping the fertility age distribution constant while varying the age-specific share of MAR births.

Interestingly, these two factors produce roughly similar effects within the ranges considered. For example, in the first column of Table 1 (constant and low age-specific MAR share), delaying childbearing alone may nearly double the share of MAR births, from 4.3% in 2023 to 8.2% in 2045–2049. This is comparable to the effect of continuing the increase in the share of MAR births at the pace observed over the past decade (first row of Table 1), which, holding the age distribution constant, would raise the share from 4.3% to 9.1% in 2045–2049.

Perhaps most striking is the combined effect of the two factors, observable diagonally in Table 1. If both factors increase only moderately—moderate increase in MAC and moderate increase in the age-specific share of MAR births—the overall share of MAR births could reach about 8% by 2045–2049. If both trends continue at the same pace observed between 2013 and 2023, the age-specific share of MAR births could exceed 15% by mid-century.

## Discussion

All projections are subject to error, and ours are no exception. However, some of the hypotheses used—whether our own or borrowed from Istat—are largely irrelevant for our results: deviations from these hypotheses have minimal impact.

Mortality illustrates this point. Higher or lower survival rates in the future will affect the number of Italian residents, particularly at older ages, but will have little effect on the proportion of MAR births. Similarly, variations in fertility rates would alter the number of births and, after roughly 15 years, the age structure of women of reproductive age. Since our projection horizon is 25 years, any variation in fertility rates would at most affect the number of women aged 15–25, and this only towards the end of the period considered here. Besides, these are ages when fertility is relatively low in Italy, and MAR very seldom used: the impact on our results would be minimal (results not shown).

Regarding migration, for simplicity and due to lack of age-specific information, we assumed that the prevalence of MAR births is identical among natives and immigrants. This is not strictly true: immigrants generally access MAR less than natives do [29], as they tend to have children at younger ages and are characterized by lower income and less familiarity with the Italian health system and bureaucracy. In theory, therefore, changes in the proportion of immigrants among potential mothers, could affect overall MAR prevalence. At reproductive ages, however, the share of immigrants in Italy is about 13% in 2023 and it is not expected to change substantially. Istat projections, on which this paper is based, allow only for a limited inflow of immigrants in the future, and naturalizations are on the rise (about 200 thousand per year recently, according to official data), which tends to reduce the share of immigrants in the population.

This leaves only two potentially relevant variables: the ongoing process of childbearing postponement and the rate of increase in MAR use. Recognizing the uncertainty surrounding both, we explored a wide range of values, including the “invariant scenario” mainly as a reference point. Historical trends—longer for MAC, shorter for %MAR—show clear, monotonic increases: MAC has been rising over decades, %MAR over the past few years. Internationally, the same pattern is observed: delayed childbearing and increased MAR use [30].

MAR practices themselves are improving [31], and the number of specialized centers has increased from 169 to 333 in the same period. These developments reinforce the tendency for greater utilization, further amplified by an “institutionalization effect”: practices once restricted to highly educated, affluent groups gradually diffuse to the wider population and become increasingly accepted [32,33], as observed with cohabitation and union dissolution [34].

Thus, the moderate increase scenario may be considered a lower bound, yielding around 8% MAR births in 2045–2049. The rapid increase scenario is not necessarily an upper bound: although theoretical limits exist (fertility cannot be delayed indefinitely, and the share of MAR births among total live births cannot exceed 100%), in the medium term these hypotheses remain plausible given current trends. Moreover, recent legislation in Italy (from January 2025) has made MAR more affordable, which is likely to further boost demand in the long term. Consequently, the rapid increase scenario may be more realistic than it seems, potentially resulting in MAR prevalence close to 15% by 2045—a very high share by current national and international standards, with potentially widespread implications.

First, both current trends and our projected scenarios point to a continued postponement of childbearing. This development may be associated, among other factors, with the increasing diffusion of MAR practices and ongoing advances in their accessibility, effectiveness, and range of applications. In Italy, this trend might further push towards a fertility schedule characterized by increasingly late ages at childbearing, extending beyond the age ranges commonly discussed in the clinical and reproductive health literature [35].

Second, MAR interventions are costly. Although we are unaware of official estimates, websites of selected Italian fertility centers suggests costs of approximately €5,000 per IVF/ICSI cycle in the private sector, although with great variability, depending on the type of intervention [36]. Considering that three interventions are usually needed per live birth, that over 17,000 MAR births occurred in Italy in 2023 [37], and that our projections indicate this number may become two to three times higher, total costs are substantial. While recent legislation shifts much of this cost from private to public providers, it does not reduce it. Furthermore, Italy’s public finances are under stress, with high deficits and a debt-to-GDP ratio currently exceeding 140% [38]. Consequently, further public subsidies may be limited, possibly introducing measures such as means-testing. Furthermore, unless additional resources are allocated to increase the number of medical staff, any future increase in MAR demand may translate into longer waiting lists, and not necessarily into successful conceptions.

Third, assisted reproduction may entail health implications: although MAR itself does not appear to increase maternal or neonatal risk, later childbearing does (Table 2). Risk increases are modest and absolute values remain low, but they highlight the importance of investing in measures that promote earlier childbearing, such as policies addressing youth economic independence, housing, and work-family reconciliation [39,40]. Allocating a substantial share of public resources to subsidize MAR, implicitly supporting delayed fertility, appears questionable, both in terms of public health and in terms of sustaining fertility.

**Table 2:** Approximate risks for selected outcomes by maternal age (various years, around 2010).

Maternal age (years)	Down syndrome at birth	Stillbirth (≥20weeks)	Preterm birth (<37weeks)	Low birth weight (<2500g)
**20–24**	**0.07%**	**0.5%**	**10–11%**	**7–8%**
**25–29**	0.08%	0.507%	9–10%	7%
**30–34**	0.14%	0.515%	10–11%	7–8%
**35–39**	0.37%	0.586%	11–12%	8–9%
**40–44**	1.0%	0.836%	14–15%	9–10%
**45+**	≥2.0%	1.325%	18–20%	11–12%

Sources: Down syndrome risk: [13]; Stillbirth: [14]; Preterm birth: [41,42]; Italy (Lombardy): [43]; Canada: [44]; Low birth weight: [41,45].

Importantly, young people in Europe deem it ideal to become parents before their 30^th^ birthday, including in Italy [46], and a high share of parents had their first child later than desired, especially among women, reaching 96.2% among mothers who had their first child at age 40–44 and 100% at age 45–49 [47].

The social implications of these trends are also substantial. Current evidence points to a continuing reorganization of life-course trajectories, characterized by the postponement and adaptations of major life transitions [48], including latest-late childbearing. This shift affects intergenerational relationships by increasing the age gap between parents and children, as well as between grandparents and grandchildren. It may also have implications for the wellbeing of individuals and couples who undergo fertility treatments in pursuit of their reproductive goals. Recent research suggests that the mental health consequences of ART may extend to both women and men, with emerging evidence indicating an elevated risk of postpartum depression among fathers following ART conception [45]. Further research is needed to examine longer-term mental health trajectories, the consequences of unsuccessful treatments, and the mechanisms linking ART experiences to parental wellbeing [49].

## Conclusions

Our projections indicate that the prevalence of births resulting from medically assisted reproduction (MAR) in Italy is set to rise sharply in the coming decades. Population ageing alone will have little impact, but the combination of continued delayed childbearing and increased use of MAR could raise the share of MAR births from 4.3% in 2023 to 11–12% by mid-century, potentially exceeding 15% under sustained trends.

These findings highlight the growing demographic and social significance of MAR in Italy. While MAR enables couples to achieve their desired family size, its expansion also reflects ongoing structural barriers to earlier childbearing. The expected surge in demand raises critical considerations for health systems, including costs, equitable access, and the capacity of medical services.

While our estimates suggest that ART may contribute substantially to births in Italy, these findings should not be interpreted as evidence that medically assisted reproduction can offset the broader structural drivers of fertility decline or compensate for the postponement of childbearing [40]. ART primarily enables individuals and couples to achieve intended births rather than increasing desired family size. Policies addressing infertility should therefore remain grounded in a rights-based reproductive health framework rather than a pronatalist one [50,51], while also recognizing the central yet often neglected role of male infertility in shaping reproductive outcomes [17,18].

Policies that focus solely on subsidizing MAR risk reinforcing the trend toward ever-later fertility. A balanced approach is needed: promoting life-course reproductive awareness among both women and men and ensuring adequate provision of MAR while simultaneously addressing the underlying drivers of delayed childbearing, such as youth economic insecurity, housing limitations, and work–family reconciliation. Only by addressing both dimensions can Italy ensure that the rising role of MAR supports, rather than substitutes for, a more sustainable fertility trajectory.
